# Drawing a Close to the Use of Human Figure Drawings as a Projective Measure of Intelligence

**DOI:** 10.1371/journal.pone.0058991

**Published:** 2013-03-14

**Authors:** Kana Imuta, Damian Scarf, Henry Pharo, Harlene Hayne

**Affiliations:** Department of Psychology, University of Otago, Dunedin, New Zealand; Universidad Europea de Madrid, Spain

## Abstract

The practice of using children's human figure drawings (HFDs) to assess their intellectual ability is pervasive among psychologists and therapists in many countries. Since the first systematic scoring system for HFDs was published in 1926, their continued popularity has led to the development of several revised versions of the test. Most recently, the Draw-A-Person Intellectual Ability Test for children, adolescents, and adults (DAP:IQ) was published. It is the most up-to-date form of HFD test designed to assess intellectual functioning across a wide age range. In the present study, we assessed the validity of the DAP:IQ as a screening measure of intelligence in both children and adults. In Experiment 1, 100 4- to 5-year-old children completed the DAP:IQ and the Wechsler Preschool and Primary Scale of Intelligence-Third Edition. In Experiment 2, 100 adults completed the DAP:IQ and the Wechsler Abbreviated Scale of Intelligence. In both experiments, we found only weak to modest correlations between scores on the DAP:IQ and the Wechsler tests. Furthermore, when we compared individual's scores on the two tests, the DAP:IQ yielded high false positive and false negative rates when screening for borderline and superior intellectual functioning. Based on these findings, and based on the lack of validity of previous HFD tests, we conclude that practitioners should not rely on HFD tests as a projective measure of intelligence.

## Introduction

Children's drawings have captured the interest of scientists since the late 19^th^ century. When tracing the developmental progression of children's drawings, early researchers found that children's drawings become increasingly more detailed and realistic as they grow older [1], [2]. For example, when children begin to draw the human figure at around 4 years of age, they typically start by drawing a “tadpole image.” That is, they represent both the head and the torso as one single figure and often represent both the arms and legs as a single pair of lines. By the time children finish preschool, they begin to differentiate the different body parts and illustrate them in their correct locations. Between 7 and 11 years of age, children begin to pay more attention to the details of the drawing such as clothing, accessories, and hair styles, typically producing realistic human figure drawings (HFDs) by the time they reach 10 to 12 years of age [3], [4], [5].

Although children's drawings become increasingly more realistic as they get older, the rate at which they develop the ability to produce realistic drawings varies from child to child. Some researchers have argued that the amount of realistic detail included in a child's drawing reflects the child's conceptual development, and therefore, children's drawings might provide a surrogate measure of their cognitive ability. Early researchers who proposed this idea used children's school work [6] or teacher ratings [7] as measures of cognitive ability and they found positive correlations between these measures and children's drawings. Later, the first systematic scoring system for children's drawings was developed by Goodenough [8] in her Draw-A-Man Test (DAMT). In the DAMT, children between 4 to 10 years of age are asked to draw a single picture of a man. Children's HFDs are scored based on the number of details in the drawing and the accuracy of the placement of each body part. Goodenough [8] claimed that her scoring system, based on conceptual elements, allows for the DAMT to be a surrogate measure of children's intelligence rather than simply a measure of aesthetic or manual skill.

Since Goodenough's [8] initial development of the DAMT, it has undergone several revisions of its procedure and scoring system. It was first revised by Harris [9] and the new test was referred to as the Goodenough-Harris Drawing Test (GHDT). The GHDT was designed to assess both children and adolescents of up to 15 years of age. In the GHDT, children are asked to draw three pictures: one of a man, one of a woman, and one of the self. In addition, a new scoring system was developed to estimate overall maturity, precision of details, and general proportion. Although Harris [9] developed scoring systems for drawings of a man and a woman, no scoring system was developed for the self-drawing. In order to address this issue, Naglieri [10] established the Draw-A-Person: A Quantitative Scoring System (DAP:QSS), in which he developed a scoring system for the self-drawing along with a composite scoring system for the three drawings. Furthermore, the DAP:QSS outlined more specific scoring criteria, provided recent norms, and increased the precision of the standard scores by providing norms for half- and quarter-year intervals [10].

Recently, a new drawing test has been developed that allows for the assessment of adults as well as children and adolescents. This new test, the *Draw-A-Person Intellectual Ability Test for Children, Adolescents, and Adults* (DAP:IQ) [11] can be used with people between 4 to 90 years of age; the DAP:IQ, therefore, is the first HFD test that can also be used with adults. In contrast to the GHDT [9] and the DAP:QSS [10] that require participants to draw three pictures, the DAP:IQ requires a single drawing of the self. There are 23 criteria for scoring the DAP:IQ including the head, hair, eyes, eyelashes, eyebrows, nose, mouth, chin, ears, neck, shoulders, arms, elbows, hands, torso, waist, hips, legs, knees, ankles, feet, clothing, and accessories. Each feature is given a score from 0 to 4, with the maximum score possible differing across items.

The ongoing development of updated forms of HFD tests points to their popularity in professional settings. Indeed, HFD tests have consistently ranked among the most popular assessment tools used by clinicians and psychologists over the past 50 years [12], [13], [14], [15], [16], [17], [18], [19]. These professionals use HFD tests as a screening device, as a test battery component, and as a proxy measure of intellectual ability. HFD tests are valued as time-efficient, non-verbal assessment tools that can be used to test children with limited attention span and language difficulties [10], [11], [20], [21], [22]. Although the versatility of HFD tests may be appealing, professionals must first consider the scientific foundation of these tests.

A number of researchers have assessed the reliability and validity of previously developed HFD tests, and the findings are highly consistent. HFD tests yield high reliability coefficients. For example, the DAP:IQ has a high level of internal consistency with Cronbach's alpha coefficients ranging between 0.73 to 0.88 [11], [22]. The inter-rater reliability and intra-rater reliability coefficients for the DAP:IQ are also high, ranging between 0.72 to 0.95 [11], [22], [23] and 0.87 to 0.97 [22], respectively. Additionally, the test-retest reliability coefficient of the DAP:IQ is reported to be 0.86 [11].

In general, HFD tests have also been found to yield modest correlations with other measures of intelligence. For example, in the DAP:IQ manual, Reynolds and Hickman [11] report that correlations between DAP:IQ scores and scores on the Detroit Test of Learning Aptitude-Primary: Second Edition (DTLA-P:2) [24] range from 0.42 (Verbal subtest) to 0.61 (Motor-enhanced subtest). Reynolds and Hickman [11] also calculated correlations between DAP:IQ scores and children's scores on the Wechsler Intelligence Scale for Children- Third Edition (WISC-III) [25]. These analyses yielded correlation coefficients of 0.33 for Verbal IQ, 0.49 for Performance IQ, and 0.46 for Full Scale IQ. Taken together, these findings suggest that the DAP:IQ is a highly reliable measure, however, its correlations to other measures of intelligence are moderate at best. Similar findings have been reported for previous HFD tests such as the DAMT [26], GHDT [26], [27], [28], [29], [30], [31], and the DAP:QSS [10], [21], [28], [32], [33], [34].

Although some experts have argued that high reliability and moderate validity support the utility of HFD tests [35], [36], it is important to note that the findings were based on large research samples. Although correlations can suggest a relation between two variables, in applied settings, these tests must correctly identify individual children who might be gifted or at risk for intellectual difficulties. Previously, a handful of studies have investigated the utility of the DAMT [26], the GHDT [26], [29] and the DAP:QSS [33], [37] as screening devices of intelligence. These studies have found that although scores on the HFD tests and well-established measures of intelligence (e.g., Wechsler scales, Stanford-Binet) are correlated, HFD tests fail to accurately identify individual children who are gifted or at risk. For example, Willcock et al. [33] administered the DAP:QSS and the Wechsler Preschool and Primary Scale of Intelligence-Revised (WPPSI-R) [38]/Wechsler Abbreviated Scale of Intelligence (WASI) [39] to 5- and 6-year-old children and found a moderate correlation between the two measures (*pr*  = 0.40, *p*<0.001). Importantly, only 3 out of 12 children who received an IQ score of 79 or below on the WPPSI-R/WASI were identified as having borderline intellectual functioning using the DAP:QSS [33]. Furthermore, 14 out of 17 children in Willcock et al.'s [33] study were misidentified as having borderline intellectual functioning when using the DAP:QSS. Given these high false positive and high false negative rates, Willcock et al. [33] argued that previous HFD tests should not be used as a surrogate measure of individual assessment of children's intelligence.

According to the examiner's manual, the recently developed DAP:IQ was designed to “improve the pervasive practice of evaluating human figure drawings as a measure of cognitive ability” (p. v) by providing up-to-date norms for assessing not only children and adolescents, but also adults [11]. To date, however, the ability of this test to adequately screen intellectual giftedness or intellectual risk has not been examined. Furthermore, given that prior drawing measures were developed exclusively for children, the screening potential of an instrument that could be used with adults has never been explored. The purpose of the present study, therefore, was to examine the validity of the DAP:IQ as a screening measure of cognitive ability in an unselected population of children (Experiment 1) as well as adults (Experiment 2).

## Experiment 1

### Methods

#### Participants

A total of 100 4- to 5-year-old children (50 male, 50 female; *M*_age_ = 4.97 years, *SD* = 0.61) were recruited from public birth records. All children were fluent English speakers and the majority were Pakeha (New Zealanders of European descent) from middle-income socioeconomic families in Dunedin, New Zealand. All children participated with written consent from their parents. Children received a small toy for their participation. This study was reviewed and approved by the University of Otago human ethics committee.

#### Procedure

All tests were administered individually by a clinical psychologist in a quiet room with desks and chairs. The order of test administration was counterbalanced across children to avoid systematic effects of warm-up or fatigue, and children were also offered breaks between the tests to prevent fatigue. There was no time limit, but the total testing time lasted approximately 30 minutes.

#### Draw-A-Person Intellectual Ability Test for Children, Adolescents, and Adults (DAP:IQ) [11]

Following the instructions in the examiner's manual, children were asked to draw the best possible picture of him or herself that was not a cartoon or stick figure. Children were instructed to draw their whole body in frontal view. There was no time limit, though most children spent approximately five minutes on their drawings. The drawings were scored and normed on the age of the child to derive standardized IQ scores according to the manual. One experimenter independently scored all of the drawings and a second experimenter scored 30% of the drawings to assess inter-rater reliability. Consistent with prior studies that have employed HFD tests, intraclass inter-rater reliability was very high (*ρ* = 0.95).

#### Wechsler Preschool and Primary Scale of Intelligence-Third Edition (WPPSI-III) [40]

A four-subtest short form of the WPPSI-III was used in the present experiment to yield estimates of children's intellectual functioning. The short form consisted of the Information (verbal), Matrix Reasoning (performance), Picture Completion (performance), and Coding (processing speed) subtests. This combination of subtests was chosen because: (a) it covers the three main areas of intelligence that are measured by the WPPSI-III (verbal, performance, and processing speed) and, (b) it has been found to be one of the most reliable (*r* = 0.96) and valid (*r* = 0.92) four-subtest combinations of the WPPSI-III [41]. In addition to the Matrix Reasoning subtest, the Picture Completion subtest was also selected from the performance area of intelligence because it is designed to tap children's visual-perceptual ability [40], which is thought to play a significant role in producing HFDs [11]. The four subtests were scored according to the guidelines set out in the WPPSI-III manual [40], and the raw scores were converted to scaled scores. The scaled scores were then converted to estimated full scale IQ scores by referring to Sattler and Dumont [41].

### Results and Discussion

Several studies in the past have found girls to outperform boys on HFD tests [33], [42], [43], [44]. Therefore, we conducted an independent samples *t*-test to assess the effect of sex on children's scores on the DAP:IQ. The *t*-test revealed that, consistent with previous studies, the girls (*M* = 110.42, *SD* = 15.42) scored significantly higher on the DAP:IQ than the boys (*M* = 102.70, *SD* = 12.86), *t*(98) = −2.72, *p* = 0.008.

**Figure 1:**
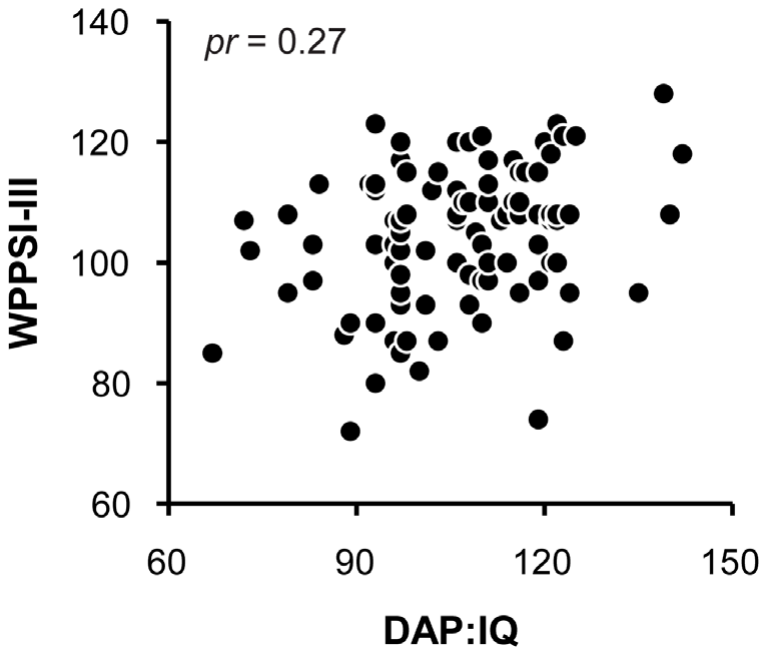
Children's results. Scatterplot showing children's standard scores on the DAP:IQ and the four-subtest short form the WPPSI-III (*pr* = 0.27).

Figure 1 shows children's standard scores on the DAP:IQ and the four-subtest short form of the WPPSI-III. Standard scores on the DAP:IQ ranged from 67 to 142 (*M* = 106.56, *SD* = 14.65) and standard scores on the WPPSI-III ranged from 72 to 128 (*M* = 104.32, *SD* = 11.45). A partial Pearson product-moment correlation, controlling for sex, was calculated to examine the relation between children's standard scores on the DAP:IQ and the WPPSI-III. This analysis yielded a positive partial correlation, *pr* = 0.27, *p* = 0.007. When the analysis was conducted without controlling for sex, *r* = 0.30, *p* = 0.002. The magnitude of this partial correlation is slightly lower than the correlations between 6- to 11-year-old children's scores on the DAP:IQ and the WISC-III that are reported in the DAP:IQ manual [11], but it is consistent with previous studies which have typically found modest to moderate correlations between children's standard scores on earlier versions of HFD tests of intelligence (e.g., DAP:QSS) and the Wechsler tests [10], [26], [27], [28], [29], [31], [32], [33], [34], [45].

**Figure 2:**
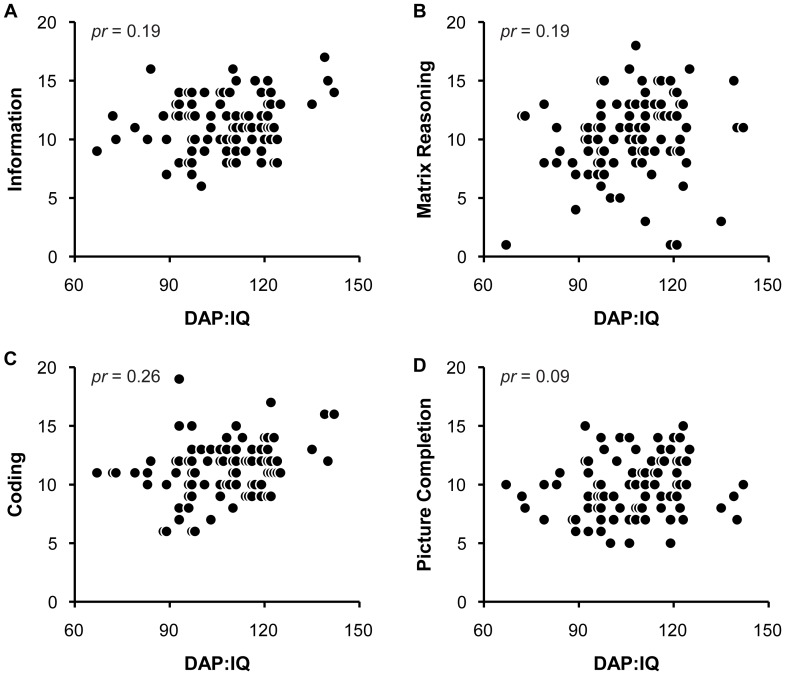
Children's correlations for each of the four WPPSI-IIIsubtests. (A) DAP:IQ and Information (*pr* = 0.19), (B) DAP:IQ and MatrixReasoning (*pr* = 0.19), (C) DAP:IQ and Coding (*pr* = 0.26), and (D) DAP:IQ and PictureCompletion (*pr* = 0.09).

In the DAP:IQ manual, Reynolds and Hickman [11] report that children's scores on the DAP:IQ correlate more strongly with their scores on the nonverbal aspects of other measures of intelligence (e.g., DTLA-P:2, WISC-III) than those on the verbal aspects. To further investigate this finding, we compared children's standard scores on the DAP:IQ with individual scores on each of the four subtests in the WPPSI-III (see Figure 2). A series of partial Pearson product-moment correlations, controlling for sex, revealed a significant, positive partial correlation between children's scores on the DAP:IQ and the Coding subtest, *pr* = 0.26, *p* = 0.01. In contrast, there were no significant partial correlations between children's scores on the DAP:IQ and the Information, Matrix Reasoning, and Picture Completion subtests (*p*>0.05).

The finding that children's scores on the DAP:IQ were significantly correlated with the Coding subtest (nonverbal), but not with the Information subtest (verbal), partially supports the idea that the DAP:IQ is “a measure of general ability but also… significantly more aligned with the nonverbal than the verbal domain” (p. 28) [11]. Children's scores on the Matrix Reasoning and Picture Completion subtests, which are also nonverbal components of the WPPSI-III, however, were not correlated with their scores on the DAP:IQ. In particular, the finding regarding the Picture Completion subtest is not consistent with the claim that visual-perceptual skills play an important role in children's performance on the DAP:IQ [11].

Lastly, we assessed the utility of the DAP:IQ as a screening measure of children's intellectual functioning. The fact that children's scores on a HFD test and a Wechsler test correlate, on its own, is not sufficient to conclude that the drawing test is an effective screen of individual children's intellectual functioning [26], [29], [33], [37]. Weak to moderate correlations between two measures often mean that one or both measures will fail to provide adequate screening power on a child-by-child basis. First, we assessed the overall hit rate of the DAP:IQ; this was done by calculating the proportion of children whose DAP:IQ classification matched that identified by the WPPSI-III. Five categories of intellectual functioning were used: Borderline (<80), Low Average (80 – 89), Average (90 – 109), High Average (110 – 119), and Superior (>120). Overall, the DAP:IQ accurately classified 36 of the 100 children into their respective categories of intellectual functioning identified by the WPPSI-III (i.e., 36% overall hit rate).

**Figure 3:**
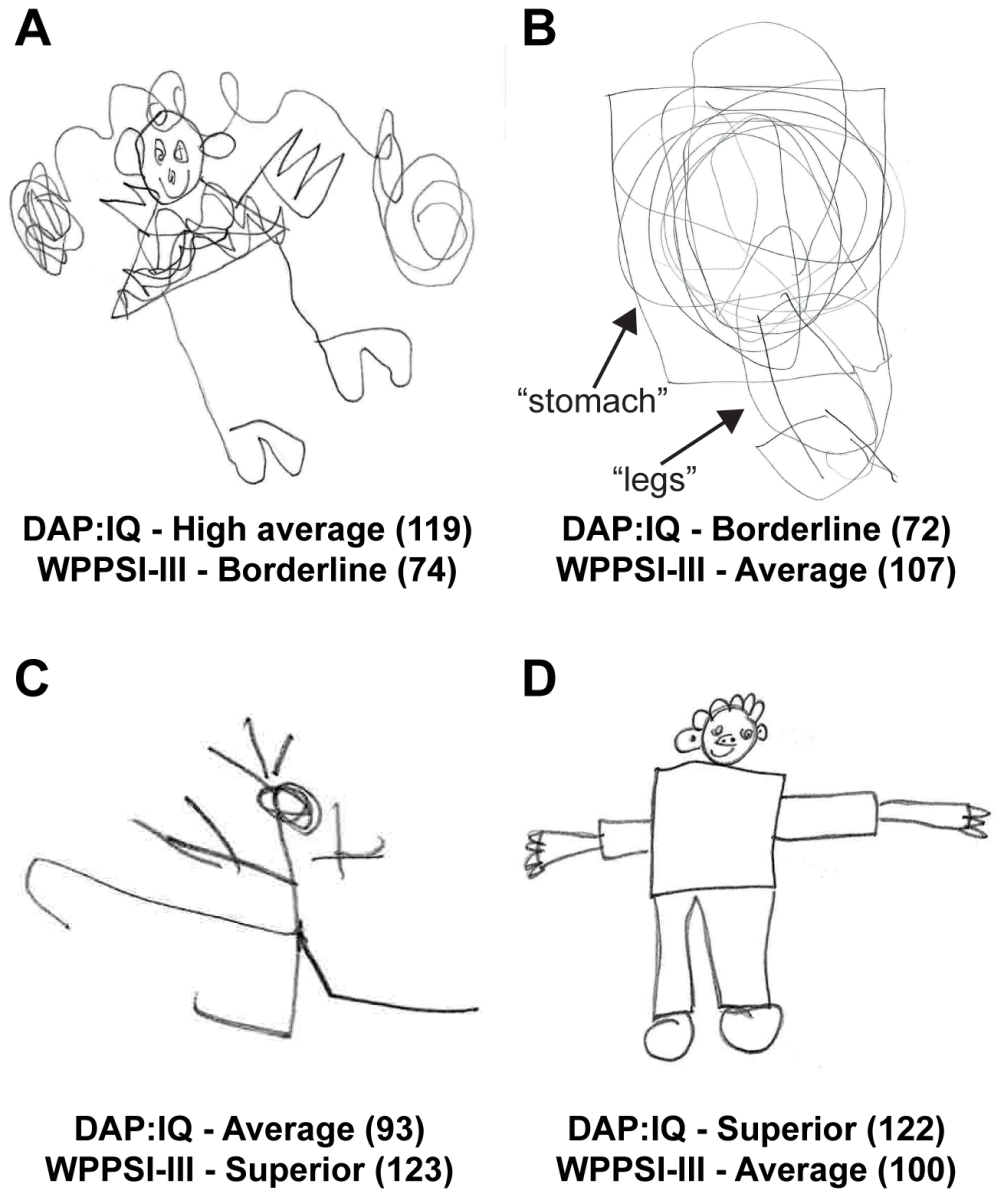
Examples of children's drawings. (A) a false negative and (B) a false positive for borderline intellectual functioning (scores below 80); and (C) a false negative and (D) a false positive for superior intellectual functioning (scores above 120). The HFD in (B) was scored according to the labels (i.e., “stomach” and “legs”) that were given by the child in the process of drawing. Children's standard scores presented within the parentheses.

Next, to assess the effectiveness of the DAP:IQ as a screening measure of children “at risk,” we examined the DAP:IQ scores of children who were identified as exhibiting borderline intellectual functioning (scores of 79 or below) on the WPPSI-III. Of the two children who obtained standard scores of 79 or below on the WPPSI-III, neither of them were identified as having borderline intellectual functioning using the DAP:IQ (i.e., 100% false negative rate). Specifically, their scores on the WPPSI-III were 72 and 74, and their respective scores on the DAP:IQ were 89 and 119. Similarly, we compared the standard scores of children who were identified as exhibiting borderline intellectual functioning on the DAP:IQ with their scores on the WPPSI-III. Of the five children who obtained standard scores of 79 or below on the DAP:IQ, none of them were identified as having borderline intellectual functioning using the WPPSI-III (i.e., 100% false positive rate; for individual scores, see Dataset S1). Figures 3A and 3B provide visual illustrations of the children's drawings that yielded a false negative and a false positive for borderline intellectual functioning, respectively.

In educational settings, the DAP:IQ is not only used to identify children at risk, but also those who are gifted [14]. Therefore, to assess the effectiveness of the DAP:IQ as a screening measure of gifted children, we examined the DAP:IQ scores of children who were identified as exhibiting superior intellectual functioning (scores of 120 or above) on the WPPSI-III. Of the 10 children who obtained standard scores of 120 or above on the WPPSI-III, 5 were not identified as having superior intellectual functioning using the DAP:IQ (i.e., 50% false negative rate; for individual scores, see Dataset S1). Furthermore, of the 20 children who obtained standard scores of 120 or above on the DAP:IQ, 15 were not identified as having superior intellectual functioning using the WPPSI-III (i.e., 75% false positive rate; for individual scores, see Dataset S1). Figures 3C and 3D provide visual illustrations of children's drawings that yielded a false negative and false positive for superior intellectual functioning, respectively.

Taken together, given that the WPPSI-III is a standardized, psychometrically sound measure of children's intelligence, these findings suggest that the DAP:IQ is not an adequate screening measure of intelligence. Despite the modest but significant correlation that was found between children's scores on the DAP:IQ and the WPPSI-III, an examination of children's individual scores on the two tests revealed the lack of ability for the DAP:IQ to correctly identify children's level of intellectual functioning.

While Experiment 1 suggests that the DAP:IQ does not provide a valid assessment of children's intelligence, it is possible that the test could be used effectively with an older population. One of the primary goals of the DAP:IQ was to develop a drawing test that was not only suitable for children and adolescents, but also for adults (ages 18 and above). To test the utility of the DAP:IQ with an adult sample, in Experiment 2, we compare adults' performance on the DAP:IQ with their performance on the WASI.

## Experiment 2

### Methods

#### Participants

A total of 100 undergraduate and postgraduate students from the University of Otago in Dunedin, New Zealand, voluntarily took part in this experiment. Adults ranged from 18 to 49 years of age (50 male, 50 female; *M*_age_  = 20.46 years, *SD* = 3.37), with 78 18 to 20 year olds, 21 21 to 30 year olds, and one person older than 31 years of age. The majority of adults were Pakeha and all were fluent English speakers. At the beginning of the experimental session, adults were asked to read an information sheet describing the experiment and to sign a consent form. Adults received course credit or a movie voucher for their participation. This study was reviewed and approved by the University of Otago human ethics committee.

#### Procedure

The procedure was identical to that described in Experiment 1, with one exception. In place of the WPPSI-III, adults were administered the WASI FSIQ-2. One experimenter independently scored all of the drawings and a second experimenter scored 30% of the drawings to assess inter-rater reliability. As in Experiment 1, intraclass inter-rater reliability was very high (*ρ* = 0.95).

#### *Wechsler Abbreviated Scale of Intelligence* (WASI) Full-Scale IQ Two-Subtest (FSIQ-2) [39]

The WASI FSIQ-2 was designed to provide an estimate of general intellectual functioning based on two of the four subtests in the WASI—the Vocabulary and Matrix Reasoning subtests. The WASI FSIQ-2 can be administered in approximately 15 minutes, and it is highly correlated with the Wechsler Adult Intelligence Scale-Third Edition (WAIS-III; *r* = 0.87) and the WISC-III (*r* = 0.81). The two subtests were scored according to the guidelines set out in the WASI manual, and the raw scores were converted to *T* scores. The *T* scores were then converted to estimated full scale IQ scores by referring to the WASI manual [39].

### Results and Discussion

**Figure 4:**
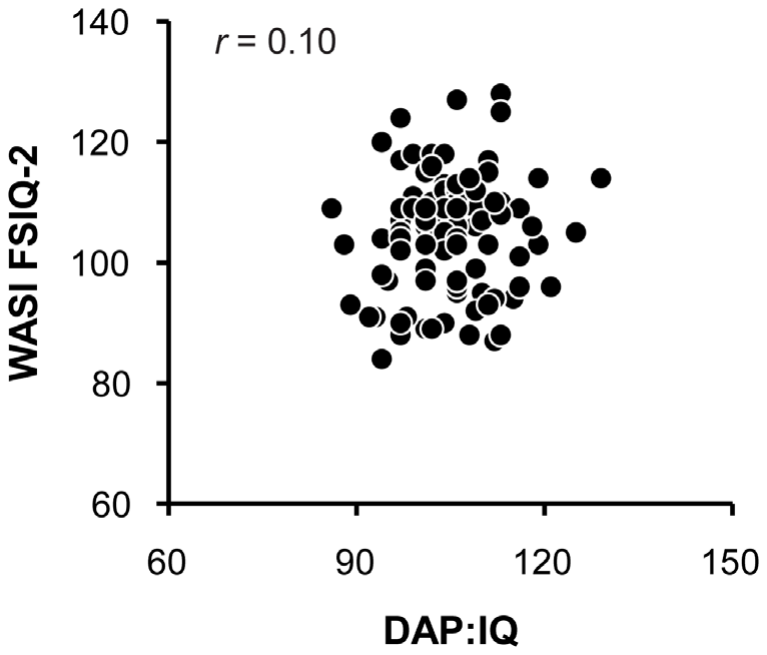
Adults' results. Scatterplot showing adults' standard scores on the DAP:IQ and the WASI FSIQ-2 (*r* = 0.10).

Preliminary analysis using an independent samples *t*-test revealed no significant effect of sex on adults' standard scores on the DAP:IQ, *t*(98) = 0.17, *p* = 0.87. To assess the relation between adults' standard scores on the DAP:IQ and the WASI, a Pearson product-moment correlation was calculated for the data presented in Figure 4. Standard scores on the DAP:IQ ranged from 86 to 129 (*M* = 104.85, *SD* = 7.71) and standard scores on the WASI ranged from 84 to 128 (*M* = 104.49, *SD* = 9.62). There was no significant correlation between adults' scores on the DAP:IQ and those on the WASI, *r* = 0.10, *p* = 0.32.

Overall, 51 of the 100 adults were classified in the same category of intellectual functioning on the DAP:IQ and the WASI (i.e., 51% overall hit rate). No adult was identified as having borderline intellectual functioning (scores of 79 or below) on the DAP:IQ or the WASI. A handful of adults, however, were identified as having superior intellectual functioning (scores of 120 or above) on the DAP:IQ and the WASI. Of the five adults who obtained standard scores of 120 or above on the WASI, none of them were identified as having superior intellectual functioning using the DAP:IQ (i.e., 100% false negative rate; for individual scores, see Dataset S2). Similarly, of the three adults who obtained standard scores of 120 or above on the DAP:IQ, none of them were identified as having superior intellectual functioning using the WASI (i.e., 100% false positive rate; for individual scores, see Dataset S2).

## General Discussion

The assessment of HFDs has long been a popular method of evaluating children's cognitive ability [12], [13], [14], [15], [16], [17], [18], [19]. To provide an improved and up-to-date HFD test of intellectual functioning that clinicians and psychologists can continue to use, the DAP:IQ was most recently created by building upon previous versions of HFD tests [11]. In Experiment 1, 4- to 5-year-old children's scores on the DAP:IQ correlated significantly with their scores on the WPPSI-III; however, the relative strength of the correlation did not differ from the modest to moderate correlations that have typically been found in the past between previous HFD tests and other measures of intellectual functioning in children from unselected populations [10], [21], [26], [32], [33]. Furthermore, an assessment of the DAP:IQ as a screening measure of intellectual functioning yielded a low overall hit rate. More specifically, the DAP:IQ both incorrectly identified children as having borderline or superior intellectual functioning (false positives), as well as failed to identify those who did have borderline or superior intellectual functioning (false negatives).

As shown in Table 1, the false positive and false negative rates that were found in Experiment 1 are comparable with the high false positive and false negative rates that have consistently been found with previous HFD tests [26], [29], [33], [37]. For Goodenough's original DAMT [8], Reisman and Yamokoski [26] found that over half of the children who were identified as having superior intellectual functioning were not identified as having the same high level of intellectual functioning by the WISC or the Stanford-Binet (see Table 1). In addition, the DAMT failed to identify the majority of children who were actually gifted. Unfortunately, the false positive and negative rates for borderline intellectual functioning is unknown for Goodenough's DAMT [8], due to the fact that Reisman and Yamokoski's [26] study only included children who had an average level of intelligence or above (score above 90). Reisman and Yamokoski [26] also tested the utility of the GHDT [9], the most up-to-date form of HFD test at the time, and found that, despite the more detailed scoring system, the GHDT was no more accurate than the original DAMT as a screening measure of gifted children (see Table 1). Significantly, Table 1 also shows that, when used with a clinical population, the GHDT still yielded relatively poor correlations that resembled those that were obtained with children who had at least an average level of intellectual functioning [26], [29]. Finally, Willcock et al. [33] investigated the utility of the DAP:QSS [10] as a screening measure of intelligence by using the same method that was used in the present study. Despite the new and “improved” scoring system that was designed for the DAP:QSS [10], the DAP:QSS yielded a similarly significant but modest correlation with the WPPSI-R/WASI, as well as high false positive and false negative rates when compared with the previous versions of HFD tests (see Table 1). Taken together, Table 1 shows that HFD tests of intelligence have consistently been found to be inadequate screening measures for both borderline and superior levels of intellectual functioning in children—Experiment 1 of the present study suggests that the most recent form of HFD test, the DAP:IQ, is no exception to this rule.

**Table 1:** Comparisons between the Four HFD Tests of Intelligence for their Correlation with Wechsler or Stanford-Binet tests, Overall Hit Rate, and False Positive and False Negative Rates for Borderline and Superior Score Classifications.

	DAMT^1^	GHDT^1,2^	DAP:QSS^3^	DAP:IQ
Correlation^4^	0.36–0.40*	0.44–0.50*/0.48–0.49**	0.40**	0.27*
Overall hit rate	54.4%^5^	50.6%^5^/*45.6%* 6	40.8%^7^	36.0%^7^
False Positive < 0	N/A	N/A/*27.2%*	82.4%	100%
False Negative <80	N/A	N/A/*76.0%*	75.0%	100%
False Positive >120	54.2%	61.9%/*75.0%* 8	62.5%	75.0%
False Negative >120	67.6%	76.5%/*97.8%* 8	63.6%	50.0%

1 Derived from Reisman and Yamokoski [26].

2 Clinical population from Aikman et al. [29] in italics; overall hit rate, false + and – rates with visual-motor functioning partialed out.

3 Derived from Willcock et al. [33]. The false + and – rates for superior intellectual functioning have not been previously published.

4 *significant at *p*<0.01, **significant at *p*<0.001.

5 Average: 90–119, Superior: >120.

6 Borderline: <80, Low average: 80–89, Average: 90–110, High average: >110.

7 Borderline: <80, Low average: 80–89, Average: 90–109, High average: 110–119, Superior: >120.

8 False + and – rates based on scores >110 for the Aikman et al. [29] data with clinical population.

One key aspect of the DAP:IQ that differentiates it from previous HFD tests is that it was designed to assess not only children and adolescents, but also adults over 18 years of age. While the DAP:IQ manual provides at least some information on the concurrent validity of the DAP:IQ as a measure of intelligence for children, it provides nothing on this with respect to adults. To date, therefore, the effectiveness of HFD tests as measures of intellectual functioning in adults has not been empirically tested. Hence, Experiment 2 represents the first empirical data on the utility of a HFD test as a measure of intellectual functioning in adults. In contrast to the modest, significant correlation that was found for children, adults' scores on the DAP:IQ did not significantly correlate with those on the WASI. Furthermore, the DAP:IQ accurately classified only half of the participants into their respective categories of intellectual functioning as identified by the WASI. More specifically, the DAP:IQ failed to accurately identify giftedness in adults. It is important to note that the high false positive and false negative rates we obtained with the adult sample may, in part, be due to the fact that a highly educated group of adults were tested. Additional studies, therefore, must be conducted to provide points of comparison for these findings, particularly by using adults from a broader range of educational backgrounds.

Despite the empirical evidence that has repeatedly challenged the validity of HFD tests, the publication of the new DAP:IQ supports the notion that HFD tests continue to be one of the most popular measures of children's intelligence that is used by clinicians and psychologists [12], [13], [14], [17]—but why?

First, HFD tests of intelligence are valued as an assessment tool that can be used with special populations [10], [11], [17], [20], [21], [22], [28]. As noted above, however, the results obtained with special populations regarding the utility of HFD tests as a screening measure of intelligence are comparably poor to those that have been obtained with normal populations (see Table 1) [26], [29]. Furthermore, the utility of HFD tests with special populations is particularly poor, even when compared to other screening measures of intelligence. For example, Prewett et al. [37] compared the validity of the DAP:QSS with that of two other screening measures of intelligence— the Matrix Analogies Test-Short Form (MAT-SF) [46] and the Kaufman Test of Educational Achievement-Comprehensive Form (KTEA) [47]—and found that, while the DAP:QSS correctly identified only 22 of the 39 developmentally handicapped (DH) children (56%), the MAT-SF correctly identified 32 of the 39 DH children (82%), and the KTEA correctly identified all 39 of the 39 DH children (100%). In addition, although several researchers have suggested the utility of HFD tests in assessing autistic children [11], [28], there are several well-known case studies in which autistic children with severe cognitive deficits have exhibited superior drawing ability [48], [49], [50], [51], [52]. These findings, together, suggest that the use of HFD tests with special populations is not warranted.

Second, when compared with other measures of children's intelligence, HFD tests are extremely quick to administer and score; the DAP:IQ, for example, can typically be administered, scored, and interpreted within 10 to 12 minutes [11]. This is perhaps the main reason HFD tests are used by practitioners who test large numbers of children. For example, educational specialists commonly utilize HFD tests of intelligence to screen children at risk and for giftedness upon school entry [14], [17]. As shown in Experiment 1, however, the use of screening devices such as the DAP:IQ for determining school entry is problematic because they yield high rates of false positives and false negatives. When children are identified as having borderline intellectual functioning, when in fact they do not (false positive), these children may be denied entry into school or placed into transitional extra-year programs, a year behind other children of the same age [14], [53]. On the other hand, when children are not identified as having borderline intellectual functioning, when in fact they do (false negative), they may not receive the appropriate intervention or preventative care that they require to succeed in school [53]. Ironically, although HFD tests are valued for their time efficiency, they are often administered as part of a test battery that may take up to or over 30 minutes to complete [14]. Considering the fact that even the quick, 2-subtest short forms of the WPPSI-III are more reliable and valid screening measures for children's cognitive abilities [41], the DAP:IQ is dispensable.

Third, while HFD tests may not be a valid measure of intelligence, they are often used as part of a test battery because the activity of drawing may help practitioners to build rapport with the children [13], [54], [55], [56], [57]. Indeed, drawing has previously been found to facilitate and enhance verbal communication with children, particularly when children are interviewed about prior experiences [58], [59], [60], [61], [62], [63]. In particular, researchers have found that drawing increases the amount of information that children provide without compromising the accuracy [58], [59], [60], [61], [62], [64]. Critically, however, this research demonstrates that it is drawing about a prior event in particular, rather than simply drawing human figures, that benefits children's verbal communication; when viewed in the light of the present study, an analysis of children's HFDs accrues little additional benefit beyond this utility. The activity of drawing, therefore, should be valued as a communication tool for practitioners working with children, whether it is to simply provide children an opportunity to talk and warm up, or to assess children's memory about past events.

The present study clearly demonstrated that the DAP:IQ [11], the most up-to-date version of HFD test, has little validity as a projective measure of intelligence in both children and adults. What is more, since the publication of Goodenough's [8] original HFD test, several revisions have been made to enhance the utility of these tests. These efforts, however, have consistently failed to produce a psychometrically sound assessment tool for children's intellectual functioning, with the DAP:IQ being no exception. Unfortunately, while children's HFDs are also commonly used to assess their personality [65], [66], [67], [68], [69], emotional well-being [70], [71], [72], and sexual abuse status [73], [74], [75], empirical evidence also stands against the utility of projective drawing tests in these domains. Given the dearth of empirical evidence supporting the use of HFDs as a projective measure, we believe it is time that practitioners draw an end to their use of children's HFD tests as a surrogate measure of children's intelligence.

## Supporting Information

Dataset S1 **Children's scores.** Individual children's scores on the DAP:IQ and the WPPSI-III.
(DOCX)

Dataset S2 **Adults' scores.** Individual adults' scores on the DAP:IQ and the WASI FSIQ-2.
(DOCX)
