# Supplementary material for: Drawing a Close to the Use of Human Figure Drawings as a Projective Measure of Intelligence
Source: PLoS One. 2013 Mar 14;8(3):e58991. doi: 10.1371/journal.pone.0058991 (PMC3597590; doi:10.1371/journal.pone.0058991)
Supplement: Dataset S1 — Children's scores. Individual children's scores on the DAP:IQ and the WPPSI-III. (DOCX) [file pone.0058991.s001.docx]

Participant DAP:IQ WPPSI-III

1 67 85

2 72 107

3 73 102

4 79 95

5 79 108

6 83 97

7 83 103

8 84 113

9 88 88

10 89 72

11 89 90

12 92 113

13 92 113

14 93 80

15 93 90

16 93 103

17 93 112

18 93 113

19 93 123

20 96 87

21 96 100

22 96 103

23 96 107

24 97 85

25 97 93

26 97 93

27 97 95

28 97 98

29 97 102

30 97 105

31 97 107

32 97 117

33 97 120

34 98 87

35 98 108

36 98 115

37 100 82

38 101 93

39 101 102

40 102 112

41 103 87

42 103 115

43 106 100

44 106 107

45 106 108

46 106 112

47 106 120

48 107 110

49 108 93

50 108 98

Participant DAP:IQ WPPSI-III

51 108 110

52 108 120

53 109 105

54 110 90

55 110 97

56 110 103

57 110 121

58 111 97

59 111 97

60 111 97

61 111 100

62 111 110

63 111 113

64 111 117

65 113 107

66 114 100

67 114 108

68 115 110

69 115 117

70 116 95

71 116 108

72 116 110

73 116 115

74 117 115

75 119 74

76 119 97

77 119 103

78 119 108

79 119 108

80 119 115

81 120 120

82 121 100

83 121 107

84 121 107

85 121 108

86 121 118

87 121 118

88 122 100

89 122 107

90 122 108

91 122 123

92 123 87

93 123 121

94 124 95

95 124 108

96 125 121

97 135 95

98 139 128

99 140 108

100 142 118
