# Supplementary material for: Drawing a Close to the Use of Human Figure Drawings as a Projective Measure of Intelligence
Source: PLoS One. 2013 Mar 14;8(3):e58991. doi: 10.1371/journal.pone.0058991 (PMC3597590; doi:10.1371/journal.pone.0058991)
Supplement: Dataset S2 — Adults' scores. Individual adults' scores on the DAP:IQ and the WASI FSIQ-2. (DOCX) [file pone.0058991.s002.docx]

Participant DAP:IQ WASI FSIQ-2

1 86 109

2 88 103

3 89 93

4 92 91

5 93 91

6 94 120

7 94 104

8 94 84

9 94 98

10 95 97

11 97 107

12 97 117

13 97 88

14 97 109

15 97 105

16 97 104

17 97 90

18 97 102

19 97 124

20 98 91

21 99 111

22 99 118

23 99 109

24 101 89

25 101 110

26 101 98

27 101 106

28 101 99

29 101 103

30 101 103

31 101 97

32 101 115

33 101 107

34 101 109

35 102 110

36 102 104

37 102 118

38 102 108

39 102 106

40 102 89

41 102 116

42 104 105

43 104 113

44 104 108

45 104 107

46 104 104

47 104 112

48 104 90

49 104 103

50 104 105

Participant DAP:IQ WASI FSIQ-2

51 104 102

52 104 109

53 104 118

54 104 90

55 106 95

56 106 96

57 106 106

58 106 112

59 106 104

60 106 110

61 106 104

62 106 113

63 106 109

64 106 97

65 106 103

66 106 127

67 108 88

68 108 114

69 109 109

70 109 112

71 109 106

72 109 92

73 109 112

74 109 99

75 109 112

76 110 95

77 110 107

78 111 117

79 111 115

80 111 93

81 111 93

82 111 103

83 112 87

84 112 94

85 112 110

86 113 128

87 113 110

88 113 108

89 113 88

90 113 125

91 115 94

92 116 101

93 116 109

94 116 96

95 118 106

96 119 103

97 119 114

98 121 96

99 125 105

100 129 114
